# Acute conversion of patient-derived Duchenne muscular dystrophy iPSC into myotubes reveals constitutive and inducible over-activation of TGFβ-dependent pro-fibrotic signaling

**DOI:** 10.1186/s13395-020-00224-7

**Published:** 2020-05-02

**Authors:** Luca Caputo, Alice Granados, Jessica Lenzi, Alessandro Rosa, Slimane Ait-Si-Ali, Pier Lorenzo Puri, Sonia Albini

**Affiliations:** 1 Development, Aging and Regeneration Program, Sanford Burnham Prebys Medical Discovery Institute, La Jolla, CA 92037 USA; 2 Present Affiliation: School of Psychology and Speech Pathology, Curtin University, GPO Box U1987, Perth, Western Australia; 3 Department of Biology and Biotechnology Charles Darwin, Sapienza University of Rome, P.le Aldo Moro 5, Rome, Italy; 4 Center for Life Nano Science, Istituto Italiano di Tecnologia, Viale Regina Elena 291, 00161 Rome, Italy

**Keywords:** Duchenne muscular dystrophy, iPSC, TGFβ, pSMAD

## Abstract

**Background:**

In Duchenne muscular dystrophy (DMD), DYSTROPHIN deficiency exposes myofibers to repeated cycles of contraction/degeneration, ultimately leading to muscle loss and replacement by fibrotic tissue. DMD pathology is typically exacerbated by excessive secretion of TGFβ and consequent accumulation of pro-fibrotic components of the extra-cellular matrix (ECM), which in turn impairs compensatory regeneration and complicates the efficacy of therapeutic strategies. It is currently unclear whether DMD skeletal muscle fibers directly contribute to excessive activation of TGFβ. Development of skeletal myofibers from DMD patient-derived induced pluripotent stem cells (iPSC), as an “in dish” model of disease, can be exploited to determine the myofiber contribution to pathogenic TGFβ signaling in DMD and might provide a screening platform for the identification of anti-fibrotic interventions in DMD.

**Methods:**

We describe a rapid and efficient method for the generation of contractile human skeletal muscle cells from DMD patient-derived hiPSC, based on the inducible expression of MyoD and BAF60C (encoded by SMARCD3 gene), using an enhanced version of piggyBac (epB) transposone vectors. DMD iPSC-derived myotubes were tested as an “in dish” disease model and exposed to environmental and mechanical cues that recapitulate salient pathological features of DMD.

**Results:**

We show that DMD iPSC-derived myotubes exhibit a constitutive activation of TGFβ-SMAD2/3 signaling. High-content screening (HCS)-based quantification of nuclear phosphorylated SMAD2/3 signal revealed that DMD iPSC-derived myotubes also exhibit increased activation of the TGFβ-SMAD2/3 signaling following exposure to either recombinant TGFβ or electrical pacing-induced contraction.

**Conclusions:**

Acute conversion of DMD patient-derived iPSC into skeletal muscles, by the ectopic expression of MyoD and BAF60C, provides a rapid and reliable protocol for an “in dish” DMD model that recapitulates key pathogenic features of disease pathology, such as the constitutive activation of the TGFβ/SMAD signaling as well as the deregulated response to pathogenic stimuli, e.g., ECM-derived signals or mechanical cues. Thus, this model is suitable for the identification of new therapeutic targets in DMD patient-specific muscles.

## Background

The identification of an effective treatment for muscular dystrophies requires reliable preclinical models of disease. In the case of Duchenne muscular dystrophy (DMD), the dystrophin-deficient mice (mdx mice) have provided a valuable model to study certain common aspects of the disease [1], but the model is unfortunately not informative on patient-specific pathological features that could only be appreciated by using “in dish” disease models from human patient-derived induced pluripotent stem cells (iPSC).

An essential pre-requisite for “in dish” models of DMD suitable for preclinical studies is the generation of an abundant, homogenous, and expandable population of patient-derived skeletal muscle cells. Although many protocols of DMD patient iPSC-derived skeletal muscles have been developed in the last years, the large majority of them require very long culture time (often over 30–40 days), as they are based on the sequential action of developmental cues [2–5] or sorting of specific populations generated by direct conversion via transgenic expression (i.e., Pax3 or Pax7) in iPSC-derived mesodermal progenies [5–8]. An alternative approach is provided by the acute conversion of DMD patient-derived iPSC into skeletal muscles by ectopic expression of MyoD in differentiating human embryonic stem cells (hESC) and iPSC that have already undergone mesodermal or mesenchymal transition [7, 9–11]. However, while MyoD-mediated conversion of somatic cells into skeletal muscle cells has been described about 30 years ago [12, 13], the extension of this technique to iPSC is complicated by the reported resistance of embryonic stem cells (ESC) to MyoD-mediated activation of skeletal myogenesis [14]. We have shown that the SWI/SNF component BAF60C (encoded by *SMARCD3*) is required for MyoD-mediated activation of skeletal muscle genes [15], and its absence confers to hESC resistance to MYOD-mediated myogenic conversion [14]. Delivery of BAF60C in hESCs enables MyoD-mediated myogenic conversion and formation of tri-dimensional contractile structures (myospheres), functionally resembling miniaturized skeletal muscles [14]. Nevertheless, it is currently unclear whether BAF60C is also required for MYOD-mediated generation of skeletal muscles from hiPSC and in particular for the establishment of an “in dish model” of DMD using patient-derived hiPSC.

Here, we provide a faster, scalable, and reproducible method to generate skeletal muscle cells directly from human iPSC, suitable for disease modeling. To this purpose, we used an enhanced version of piggyBac (epB) transposable vectors and epB MyoD [16] that increase transposition efficiency in human pluripotent stem cells. Transgene expression was kept under a TET transactivator gene control, in order to achieve inducible expression of MYOD and BAF60C in hESC and iPSC and to allow a straightforward and rapid myogenic differentiation.

We exploited our method to derive skeletal muscle cells from control and DMD iPSC, as an “in dish” model of DMD pathogenesis. We show that constitutive and inducible deregulation of TGFβ-SMAD2/3 pro-fibrotic network—a key component of DMD pathogenesis [3, 17–19]—is recapitulated in our system and can be used to screen for compounds with therapeutic potential in DMD.

## Results

### Direct generation of skeletal muscle cells from hiPSC using inducible MyoD and BAF60C enhanced piggyBac vectors

**Fig. 1:**
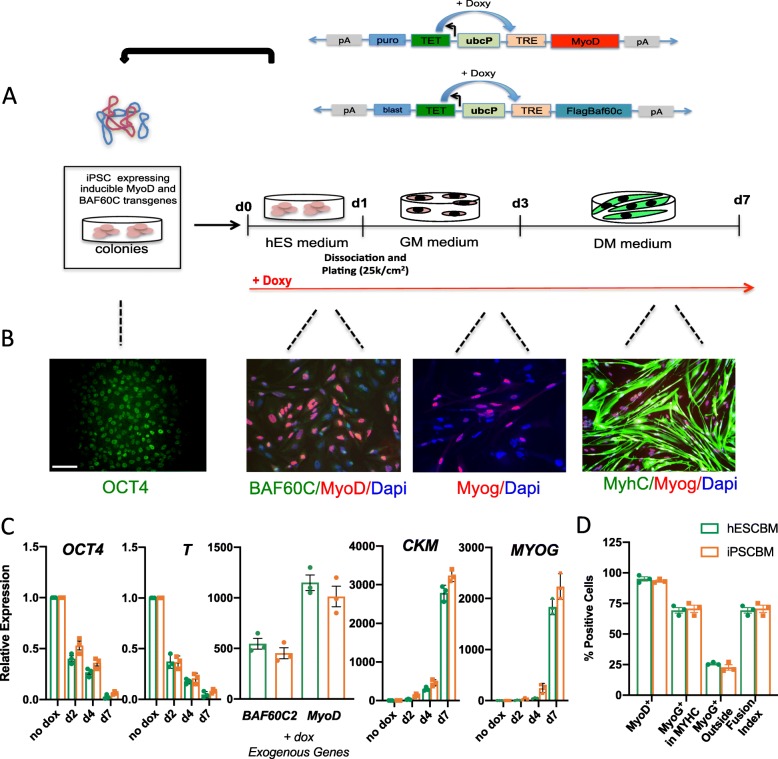
Generation of stable hESC and iPSC lines and myogenic differentiation protocol. **a** Top: Scheme of inducible MyoD and Baf60c expression vectors generated using the enhanced version of piggyback (ePB) as described [16]. Puro, puromycin resistance; Bsd, blasticidin resistance; UbcP, ubiquitin C constitutive promoter; TRE, Tet-responsive element. Bottom: Protocol for myogenic differentiation of puromycin and blasticidin-resistant cells (hESC or iPSC^BM^) obtained after stable integration of the ePB vectors. Differentiation protocol starting from hESC^BM^ and iPSC^BM^. Transgene expression is achieved by the addition of doxycycline (doxy, red line) in the hES medium for 24 h. Myogenic conversion is then triggered by switch to the proliferation medium (GM) at day 1 and to the differentiation medium (DM) at day 3. **b** Representative immunofluorescence images of hESC^BM^ or iPSC^BM^ at each step of the protocol. iPSC^BM^ at the pluripotent stages are marked by OCT4, followed by the expression of BAF60C (green) and MyoD (red) upon doxy induction at d1 and the appearance of MYOGENIN at d3. At day 7, myotubes are visible and express the marker of terminal differentiation myosin heavy chain (MYHC, green) containing MYOGENIN-positive nuclei (Myog, red). Nuclei are counterstained with DAPI. Scale bar, 50 μm. **c** Relative gene expression by qRT-PCR of the indicated genes in a time course. Data are represented as average ± SEM (*n* = 3). *p < 0.05, **p < 0.01, ***p < 0.001 (unpaired Student’s *t* test). **d** Quantification of markers of myogenic conversion efficiency (e.g., MyoD, Myog, MyHC) in MyoD/BAF60C expressing hESC and hiPSC after 7 days of culture in DM (as average ± SEM)

We generated homogeneous cultures of human skeletal myotubes from DMD (or control) patient-derived iPSC, by acute conversion via ectopic expression of MyoD and BAF60C. We delivered into our iPSC cultures the epB transposon vectors for MyoD and BAF60C by electroporation and subsequent selection for BAF60C and MyoD vector-containing cells (Fig. 1a) to generate stable cell lines that could differentiate into skeletal myoblasts by sequential exposure to different culture conditions. At day 0 (d0), induction of the transgenes was achieved by addition for 24 h of doxycycline (doxy) at 200 ng/ml in pluripotent cells maintained in the hESC culture medium (expressing pluripotent markers such as OCT4) (Fig. 1b). At day 1 (d1), cells were dissociated in single cells and plated at 25 k/cm^2^ in the growth medium (GM) for 2 days in the presence of doxy. At day 3 (d3), cells were placed in the differentiation medium (DM) that contains N2 supplement, which was shown to improve myogenic differentiation [2] (Fig. 1b). Upon induction of BAF60C and MyoD (Fig. 1b, c), iPSC sequentially expressed early (myogenin) and late (muscle creatine kinase; CKM) myogenic markers (Fig. 1c). The activation of the myogenic program was accompanied by the downregulation of pluripotency genes (OCT4) and the mesodermal marker Brachyury T (T) (Fig. 1c), consistent with the ability of MyoD to “erase” the cell of origin transcriptome profile, upon commitment to the myogenic lineage [20]. Overall, by day 7, around 60 to 70% of the BAF60C/MyoD-expressing hiPSC (iPSC^BM^) could phenotypically differentiate into multinucleated skeletal myotubes (Fig. 1d). Consistently, qRT-qPCR analysis detected induction of markers of terminal differentiation, such as *MYOGENIN* (*MYOG*) and *Creatine Kinase*, *Muscle* (*CKM*) in these cultures (Fig. 1b, c), and immunofluorescence revealed the simultaneous expression of *MYOG* and Myosin Heavy Chain (MyHC) proteins in myotubes. We could also observe remaining mononucleated MYOG-positive cells outside the myotubes, indicating that more than 90% of the cells entered the myogenic program. By contrast, the absence of Pax7-positive cells indicates that this experimental system is not suitable for the study of reserve muscle stem cells.

### Generation of skeletal muscles from DMD patient-derived hiPSC leads to aberrant activation of TGFβ1-SMAD signaling

We exploited the strategy described above to directly generate skeletal myotubes from DMD patient-derived hiPSC, as compared to their healthy counterpart. We used two different human healthy (control) and DMD hiPSC lines as described in the “Material and methods” section. DMD hiPSC lines carry different types of mutations in the *DYSTROPHIN* gene: a stop codon mutation at exon 59 (DMD iPSC^ex59X^) that we used for the majority of the experiments shown in the main figures, and exon 45 deletion (DMD iPSC^ex45del^).

**Fig. 2:**
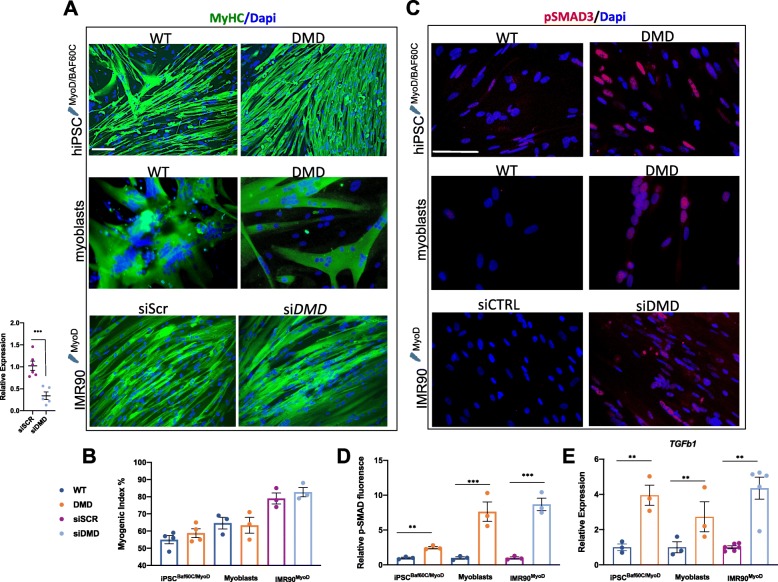
Evaluation of the myogenic potential of DMD iPSC^BM^ and activation status of TGFβ signaling. **a** Immunofluorescence for MyHC, performed in cells differentiated into myotubes. Control and DMD iPSC (ex59X), expressing inducible ePB vectors for MyoD and BAF60C (iPSC^BM^), were differentiated according to the protocol described in Fig. 1. Control and DMD human myoblasts were differentiated by medium switch; IMR90 cells, previously nucleofected with inducible ePB vector for MyoD (IMR90^MyoD^), were differentiated by medium switch (see the “Materials and methods” section for details). To monitor the effect of the lack of *DYSTROPHIN* during myogenic conversion in IMR90, cells were transfected both in GM and DM conditions with siRNAs for *DYSTROPHIN* (siDMD) or a scrambled sequence (siScr). The level of *DYSTROPHIN* downregulation is shown on the left. **b** Myogenic index (bottom graph) was calculated by immunofluorescence staining, as the percentage of nuclei within MyHC-expressing myotubes. **c** Immunofluorescence for pSMAD in iPSC^BM^, human myoblasts, and IMR90^MyoD^-derived myotubes. Nuclei were visualized by DAPI. Scale bar, 50 μm. **d** The percentage of pSMAD3-positive cells was calculated counting 10 fields and using the same exposure parameters among the samples. **e** TGFβ1 relative expression monitored in all the cells. The expression levels are shown as relative to the control or siSCr. Data are represented as average ± SEM (*n* = 3) *p < 0.05; **p < 0.01; ***p < 0.001 (unpaired Student’s *t* test)

To induce differentiation of DMD iPSC-derived myoblasts, we cultured them at high confluence, as we observed that low confluence would partially affect their myogenic potential (data not shown), possibly because of the inhibitory effect of TGFβ on myoblast differentiation at low confluence. Under these conditions, DMD iPSC^BM^-derived myotubes were phenotypically indistinguishable from control iPSC^BM^-derived myotubes and expressed similar levels of myogenic markers (Fig. 2a, b, S1 and S2A). Consistently, DYSTROPHIN deficiency did not impair the ability of DMD-derived primary myoblasts to differentiate into MyHC-expressing multinucleated myotubes (Fig. 2a, b) (this issue is further discussed in the “Discussion” section). Likewise, siRNA downregulation of *DYSTROPHIN* did not compromise the formation of multinucleated myotubes from MyoD-converted human IMR90 fibroblasts (Fig. 2a, b). Of note, in all cases, *DYSTROPHIN*-deficient myotubes invariably exhibited constitutive activation of TGFβ signaling, as shown by the increased nuclear signal of the TGFβ nuclear effectors phospho-SMAD2/3 (using an anti-pSMAD3 antibody) (Fig. 2c, d, S2B) and the increased expression of *TGFβ1* itself (Fig. 2e).

### High sensitivity to TGFβ1-SMAD signaling activation in skeletal muscle generated from DMD patient-derived hiPSC

**Fig. 3:**
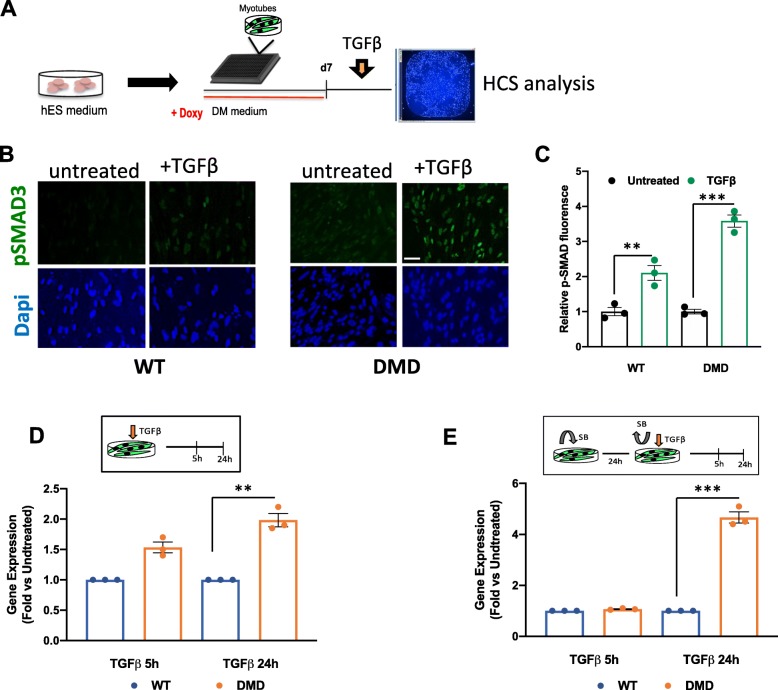
Employment of control and DMD iPSC in high-content screening setting for the assessment of TGFβ response. **a** Schematic representation of the protocol used for HCS. To ensure higher control over cell number and reproducibility, cells have to be dissociated in single cells before doxycycline treatment. Cells are plated in Matrigel-coated 384-optical well plates, fixed, and stained and then analyzed by MetaXpress Analysis software. **b** Representative immunostaining of control and DMD iPSC (ex59X)-derived myotubes, stained for pSMAD3 in normal condition and upon treatment with TGFβ (20 ng/ml) and **c** quantification of the percentage of pSMAD3 fluorescence obtained by MetaXpress Image analysis software, setting background and size parameters as described in the “Materials and methods” section. Data are expressed as fold change. **d** Gene expression of *TGFβ1* in control and DMD iPSC-derived myotubes expressed as fold induction of TGFβ-treated versus untreated cells. **e** Gene expression of *TGFβ1* mRNA expression in control and DMD iPSC-derived myotubes pre-treated with TGFβR1 inhibitor (SB-431542 (10 μM)), expressed as fold induction of TGFβ-treated versus untreated cells. All data are represented as average ± SEM (*n* = 3). *p < 0.05; **p < 0.01; ***p < 0.001 (unpaired Student’s *t* test)

We further investigated whether skeletal muscles generated from DMD human patient-derived iPSC could be more susceptible to the activation of SMAD2/3 signaling as compared to their normal counterpart, when challenged with the exposure to recombinant TGFβ1. We employed the high-content screening (HCS) platform to detect aberrant SMAD2/3 signaling (Fig. 3a). Myogenic differentiation was achieved according to the protocol illustrated in Fig. 1 (see details in the “Materials and methods” section). At d7, myotubes were treated with TGFβ, and the analysis of nuclear pSMAD3 was performed by immunostaining at different time points from the treatment. Nuclear pSMAD3 was quantified using ImageXpress Micro High Content Screening System, and MetaXpress Analysis software (Molecular Devices), which allows accurate quantification of the signal thanks to a “mask-assisted” selection of nuclear signal, defined by the tuning of specific parameters, such as signal background and size (Fig. S3). This quantification results in a very accurate background subtraction that cannot be reached by the common software analysis. We used two different antibodies that recognize pSMAD2/3 as a readout of the activation of TGFβ-SMAD signaling in DMD myotubes, to support the reproducibility of this assay (Fig. S3). Exposure to recombinant TGFβ (20 ng/ml) in the culture medium for 5 h and 24 h led to nuclear accumulation of pSMAD2/3 quantified as a percentage of pSMAD3 fluorescence at 24-h treatment in both DMD and control hiPSC-derived myotubes. However, DMD hiPSC-derived myotubes showed a 2-fold increase in nuclear accumulation of pSMAD2/3 in response to TGFβ, as compared to control myotubes (Fig. 3b, c, S2B). Likewise, DMD hiPSC-derived myotubes showed increased levels of *TGFβ1* transcripts 24 h after the exposure to recombinant TGFβ (Fig. 3d). To rule out the possibility that higher TGFβ response observed in DMD myotubes was due the higher basal levels of pSMAD2/3 (Fig. 2c–e), we pre-treated control and DMD hiPSC-derived myotubes with the SB-431542 ALK5 (TGFβRI) inhibitor 24 h prior to the exposure to TGFβ (Fig. 3e). The inhibition of autocrine activation of TGFβ pathway was revealed by the drastic reduction of the nuclear pSMAD3 signal in DMD myotubes (S4A). TGFβ response was also monitored by measuring *TGFβ1* expression with RT-qPCR, which showed a delayed but greater response to TGFβ in DMD myotubes pre-treated with SB (5-fold in DMD vs. control myotubes) (Fig. 3e).

This evidence demonstrates that DMD myotubes exhibit an aberrant activation of the TGFβ pathway, in addition to the constitutive activation, implying that autocrine secretion of TFGβ is not the sole source of over-activation of pro-fibrotic response in DMD myotubes. Moreover, we tested the suitability of the system as a cell-based assay for drug screenings by using pharmacological inhibition of pSMAD. For this purpose, we used the “dual-SMAD” inhibitor cocktail reported in Choi et al. [3] composed by LDN193189 and SB431542 and we evaluated the dose-response effect by HCS. We used the cocktail at increasing concentrations (Fig. S4B) and observed a gradual decrease in the nuclear pSMAD2/3 signal in myotubes, as compared to control cells treated with the vehicle (DMSO), indicating a dose-dependent response to pharmacological inhibition of pSMAD (Fig. S4B-C).

### Electrical pacing-induced contractions trigger TGFβ activation and exacerbate the fibrotic pathway in DMD muscles

We further assessed whether our “in dish” model of DMD could be exploited to study TGFβ response and activation of pro-fibrotic process signaling in myotubes within a more physiological context, by evoking myotube contraction with prolonged time of electrical pacing. We sought to use this system to recapitulate the key pathogenic mechanism of DMD, such as the biomechanical stress-dependent inflammatory and fibrotic response.

**Fig. 4:**
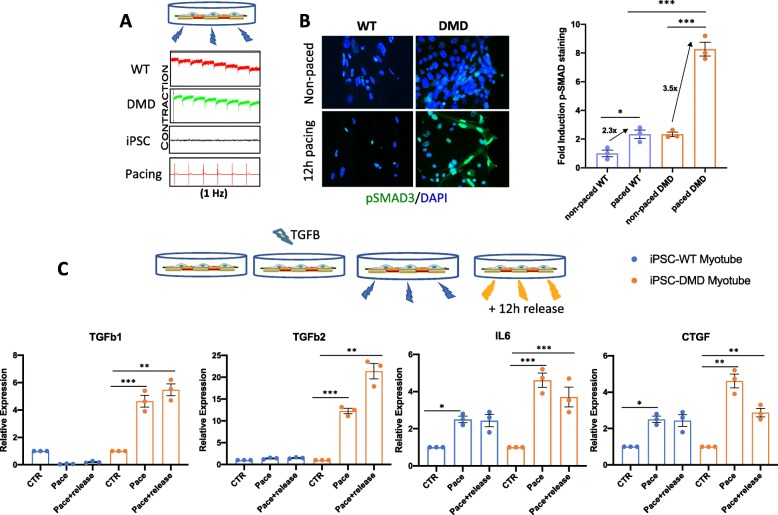
Pacing-induced contraction in iPSC-derived myotubes and activation of pSMAD signaling. **a** xCELLigence® RTCA CardioECR System (Acea Biosciences) was used to pace the cells electrically for 12 h, using 1 Hz, 0.5 ms pulse length, and 2.2 volt intensity. Contraction profiles are shown for each cell line including the negative control (non-excitable cell iPSC). The *Y* axis represents cell index and the *X* axis represents time. The contraction profiles indicate that iPSC-derived myotubes can contract after pacing. **b** Immunofluorescence for pSMAD3 in control iPSC- and DMD iPSC (ex59X)-derived myotubes in control conditions (non-paced) or after 12 h pacing and quantification of pSMAD3 accumulation, expressed as a percentage of pSMAD fluorescence of paced versus non-paced cells. *p < 0.05; **p < 0.01; ***p < 0.001 (unpaired Student’s *t* test). **c** Activation of pro-fibrotic genes (*TGFβ1*, *TGFβ2*, *IL6*, and *CTGF*) was monitored in control and DMD iPSC-derived myotubes after 12 h pacing (paced), after 12 h release from pacing (paced + release) and compared to control not paced cells (Ctr). The conditions used allow to see a greater and persistent fibrotic response in electrically paced DMD iPSC-derived myotubes as compared to control iPSC-derived myotubes. Data are represented as average ± SEM (*n* = 3). *p < 0.05; **p < 0.01; ***p < 0.001 (unpaired Student’s *t* test)

To this purpose, we used the state-of-the-art pacing system xCELLigence® RTCA CardioECR System (provided by Acea Biosciences), which allows simultaneous measurement of myocytes contractility and electrophysiology. This platform, originally designed for cardiomyocytes, was adapted to cell types competent to respond to electrical pacing (i.e., myotubes) and allowed the synchronization of contractions. While cardiomyocytes spontaneously contract, iPSC-derived myotubes did not display this feature (although we could detect sporadic spontaneous contractions). For this reason, we electrically stimulated DMD and control iPSC-derived myotubes for 12 h, under different parameters. We could reproducibly detect myotube contractions by pacing the cells for a prolonged time of 12 h at 1 Hz, with 0.5 ms, and recorded contraction events in all the replicates (Fig. 4a), as shown in the graphs (red = control, green = DMD, *X* axis = time, *Y* axis = cell index). The contraction signals were compared to those of control-undifferentiated iPSC, which are not excitable by electrical pacing. Accumulation of pSMAD2/3 was monitored by immunofluorescence and quantified using Image J software and values are reported as fold change of pSMAD3 fluorescence upon pacing, as compared to control non-paced cells (Fig. 4b). Again, we detected pSMAD3 nuclear accumulation following electrical pacing at a greater extent in DMD iPSC^ex59X^-derived myotubes (3.5 fold) as well as in DMD iPSC^ex45del^, when compared to control myotubes (2.3 fold), indicating that pacing-induced contraction is sufficient to trigger an exacerbated activation of TGFβ/SMAD signaling in DMD myotubes (Fig. 4b and S2C). We next monitored the activation of pro-fibrotic genes (*TGFB1*, *TGFB2*, *IL6*, and *CTGF*) in control and DMD iPSC-derived myotubes post-contraction, by evaluating their expression profile right after 12 h of pacing (paced) and after 12 h release from pacing (paced + release) and compared this profile to the non-paced myotubes (control) (Fig. 4c). A greater and persistent increase in the expression of fibrotic genes was observed in electrically paced DMD myotubes, as compared to control myotubes (Fig. 4c). The activation and persistence of pro-fibrotic gene activation were also evaluated and validated in human immortalized myoblasts induced to differentiate and then paced using the same conditions of pacing applied for iPSC-derived myotubes (Fig. S5). Of note, we could observe that in control iPSC-derived myotubes, the electrical pacing did not result in a significant activation of the TGFβ/SMAD pathway, as compared to chemically induced TGFβ activation, unlike DMD-derived myotubes (Fig. 4 and S5). This evidence suggests that, in our conditions, electrical pacing could evoke contraction-dependent pathogenic events typical of DMD muscles, such as susceptibility and consequently activation of TGFβ and pro-fibrotic genes.

## Discussion

The identification of the molecular mechanisms driving hESC and disease-specific hiPSC differentiation into multiple lineages is of key importance in regenerative medicine. Besides the potential to generate patient-specific cell populations for gene corrections and transplantations, the availability of patient-derived iPSC provides the unprecedented opportunity to recapitulate the disease in a dish and therefore to use it as a screening platform to find new therapeutic targets involved in the pathogenesis or novel compounds able to revert a disease phenotype.

Here, we report a fast, simple, and efficient method for acute iPSC conversion into muscle cells based on the use of transposon-based vectors encoding two key factors, MyoD and BAF60C, that guarantee a straightforward conversion of iPSC towards myogenic cells.

We show that this system is amenable to model DMD and could be suitable for a cell-based high-content screening-based drug discovery for DMD. Studies aimed at the identification of nodal pathways implicated in DMD development and disease progression downstream to DYSTROPHIN have revealed that TGFβ triggered pro-fibrotic network is invariably activated in DMD muscles, irrespective of the *DYSTROPHIN* mutation [3, 18]. In particular, nuclear accumulation of the active, phosphorylated form of SMAD 2/3 (phospo-SMAD2/3) has been observed in DMD [3]. Indeed, increasing evidence points to fibrosis as a key pathogenic determinant of DMD progression, being responsible for the loss of contractile fibers, but also contributing to the progressive impairment of the regeneration ability of DMD muscles [19, 21, 22]. As such, targeting pathogenic events downstream of DYSTROPHIN, as the molecular networks implicated in the development of fibrosis and related maladaptive responses, are emerging as promising therapeutic interventions that can effectively counteract disease progression in DMD patients [23].

Our protocol allows efficient myogenic conversion of both control and DMD iPSCs, and we show that DMD iPSC-derived myotubes display an activated SMAD signaling in unperturbed conditions. Moreover, DMD iPSC-derived myotubes also exhibit an exacerbated response to TGFβ that can be reliably quantified by pSMAD nuclear accumulation either in response to recombinant TGFβ or upon electrophysiological stimulation. The system reported herein has therefore the potential to be used as a screening platform for the discovery of compounds that target TGFβ-activated responses in dystrophic muscles.

In a recent study, Choi et al. showed that DMD hiPSC-derived muscles, irrespectively of the mutation, exhibit higher nuclear pSMAD2/3 signal compared to controls and that aberrant SMAD activity was associated with impaired fusion of DMD hiPSC-derived myoblasts, since anti-TGFβ compounds that lowered nuclear pSMAD2/3 levels could also correct the fusion defects. We did not observe an impaired differentiation into multinucleated myotubes in DYSTROPHIN deficient myoblasts generated from DMD hiPSC, from DMD myoblasts, or from MyoD-converted human fibroblasts. While this discrepancy is likely due to the different protocols used (transcription factors vs. small molecules) for the induction of skeletal myogenesis in hiPSC, the similar ability to differentiate into myotubes exhibited by DMD and control myoblasts indicate that additional parameters could account for this discrepancy. Among them, we suggest that high cell confluence could be a key determinant for tolerance to TGFβ-mediated inhibition of differentiation, as we found that low confluence compromises the differentiation ability of DMD hiPSC-derived myoblasts. Regardless, we observed that inhibition of the TGFβ/BMP4 pathway could further improve the myogenic potential of both control and DMD iPSC. This is consistent with the reported lower fusion index of iPSC-derived myoblasts, as compared to adult primary human myoblasts, due to elevated TGFβ levels in the iPSC cultures [3, 24].

The importance of having generating contractile myotubes from patient-derived iPSC resides in their potential exploitation for “in dish” model of DMD, by performing functional analysis of their biomechanical properties. We found that both control and DMD iPSC-derived myotubes responded to contractile stimuli, and we were able to recapitulate the fibrotic pathological outcome of DMD, as revealed by a pSMAD2/3 accumulation in the nucleus and by the higher and persistent activation of downstream pro-fibrotic target genes. Overall, the method proposed to generate muscle cells from patient-specific iPSC is particularly suitable to study aberrant activation of pathogenic pathways (i.e., TGFβ and other fibrotic signaling) in DMD muscles and can be exploited to investigate the molecular basis of DMD and the identification of molecular targets for interventions that counter the fibrogenic activity of DMD muscles.

In this regard, it is worth noting that pacing-induced contraction in vitro elicited upregulation of IL6 transcripts only in cultures of DMD myotubes, with only a modest increase in IL6 observed in control myotubes. While this seems in apparent conflict with previous works reporting on IL6 release from muscles during exercise/physical activity [25], we argue that muscle contraction within the context of systemic exercise in vivo or upon electrical stimuli in vitro differs on multiple parameters that might account for the different output of IL6. Indeed, exercised muscles in vivo contain cell types (i.e., macrophages, FAPs) that could contribute to the output of IL6 secretion and that are not present in pure cultures of iPSC-derived skeletal myotubes. Furthermore, pacing-induced contraction of iPSC-derived skeletal myotubes does not account for the complexity of exercise-mediated contraction of skeletal muscles that typically includes multiple rounds of contraction at different frequencies and is mediated by neuromuscular junctions, which were not included in our in vitro system. Finally, within the context of exercise, skeletal muscles are exposed to systemic factors that are obviously not present in cultures of iPSC-derived myotubes. While we acknowledge that the absence of NMJ and systemic factors currently represent a limitation in the interpretation of data derived from “in dish” models of DMD, we also note that an intrinsic advantage of our experimental system with patient-derived cultures of myotubes is the opportunity to study the specific effect of DYSTROPHIN deficiency on the transcriptional output from skeletal myofibers, regardless of the complexity of the systemic environment in vivo. This might help to understand the primary molecular relationship between DYSTROPHIN deficiency and altered profiles of gene expression in skeletal myofibers in response to contraction, in order to identify specific targets for the correction of the pathogenic transcription output in DMD muscles. In this regard, our data provide the first evidence that contraction stimulates *IL-6* expression (as well as the expression of other fibrogenic genes) in DMD myotubes, but not in their control counterpart, regardless of the influence of secondary events from non-muscle cells (i.e., chronic inflammation, fibrosis) that are typically observed in DMD patients.

## Materials and methods

### Cell lines

The human ES cells used are H9 (from the Stem Cell Core), Control iPSC (iPSC-control) were obtained from La Spada Lab (UCSD) from healthy patient’s fibroblasts (IRB #130337ZF) or from Coriell (AG08C5). Duchenne Muscular Dystrophy iPSC (iPSC-DMD) was obtained from DMD patient’s fibroblasts (Coriell GM04619) carrying mutation in stop codon at 2905 of exon 59. iPSC lines were generated by Sendai Virus reprogramming (SBP Lake Nona). DMD iPSC (Coriell GM25313) carries deletion at exon 45. Both hESC and iPSC were maintained and propagated as colonies in hES maintenance medium mTeSR1 and passaged using ReLeSr (Stem Cell Technologies) on Matrigel-coated wells (BD Bioscience).

IMR90 cells (obtained from Coriell) were cultured, nucleofected, and differentiated as described [26]. siRNA transfection was performed using siRNAs against a scrambled sequence (control) or *DYSTROPHIN* (DMD) (Dharmacon) at 20 nM concentration with RNAiMAX, first in proliferating cells and then 1 h after differentiation.

Human control and DMD myoblasts (carrying deletion of exons 45-52), obtained from AFM-MyoBank, were cultured in Skeletal Muscle Cell Growth Medium Kit (Promocell), and differentiation was induced by switching the medium to DMEM (Invitrogen) supplemented with insulin (10 μg/ml). Recombinant human TGFβ1 (Peprotech) was used at 20 ng/ml. SB-431542 (MedChemExpress) and LDN-193189 (Selleckchem) were added for 24 h in cell media.

### Plasmid construction and generation of stable cell lines

The epB-Puro-TT-mMyoD plasmid is described in Lenzi et al. [16]. Briefly, the epB-Puro-TT-hBaf60c was obtained through the subcloning of the FLAG-hBAF60c2 cDNA (from the pBMN-FlagBaf60c2 vector) into the epB-Bsd-TT plasmid (Bsd = blasticidin resistance) [27]. Both vectors are endowed with a tetracycline-responsive promoter (TRE) that drives the expression of the transgenes. MyoD construct contains the constitutive puromycin resistance cassette fused to the rtTA (TET transactivator) gene, whereas Baf60c construct contains a Blasticidin resistance cassette fused to the rtTA.

The plasmids were transfected into the cells by electroporation using the Neon Transfection System, following the manufacturer’s instructions. epB-Puro-TT-mMyoD, epB-Bsd-TT-hBaf60c, and the transposase were transfected at the ration 2:2:1 respectively, using a total of 5 μg of DNA for 1 × 10^6^ cells. Selection was performed using 1.5 μg/ml of Puromycin and 3 μg/ml of Blasticidin at the same time.

### Myogenic differentiation of hESC and iPSC

hESC or iPSC stably expressing MyoD and Baf60C inducible transgenes (hESC^BM^ or iPSC^BM^) were propagated in mTeSR1 on Matrigel-coated wells. To induce myogenic differentiation starting from iPSC colonies, doxycycline (200 ng/ml) was added in cells maintained in mTeSR1 (day 0). After 24 h of treatment with doxycycline (day 1), cells were dissociated as single cells using TripLe and plated 25 k/cm^2^ in GM medium (knockout DMEM (Invitrogen) supplemented with 1 mM l-glutamine, 20% knockout serum replacement medium (KOSR, Invitrogen), 1 mM sodium pyruvate, 0.1 mM nonessential amino acids (NEAA, Invitrogen), 50 U/ml penicillin, 50 mg/ml streptomycin (Invitrogen), 0.1 mM beta-mercaptoethanol (Invitrogen)) plus hES cell recovery supplement (10 uM) (Stemgent) and doxycycline.

On day 3, GM medium was switched to DM medium (DMEM containing 2% Horse Serum and 1× ITS supplement (SIGMA)) and doxycycline until day 7. To induce myogenic differentiation starting from single cells, hESC^BM^ or iPSC^BM^ were first dissociated into single cells, plated at 15 k/cm^2^ density, and then exposed to doxycycline in mTesR1 medium (d0).

### Immunofluorescence

Cells grown on Matrigel-coated 384-well optical tissue culture plates (Greiner Bio-One) were fixed with 4%PFA for 15 min at room temperature, and permeabilized in 0.5% Triton X-100 then blocked in 4%BSA (blocking buffer) for 30 min. Incubation with the primary antibodies diluted in blocking buffer was performed overnight. The antibodies used are as follows: anti-Myod (BD Bioscience, 554,130), anti-Myogenin (clone F5D, DSHB), anti-GFP (Invitrogen, A11122), anti-Baf60c custom made, anti-myosin heavy chain (MF20, DSHB), anti-pSMAD 2/3 (cs #8828 s, Cell signaling) and anti-pSMAD3 (ab52903, abcam), desmin, Ki67, and Fast MyHC (abcam). Alexa-488 or 555 or 647 were used as secondary antibodies (Invitrogen). Nuclei were counterstained with DAPI.

High-content imaging analyses were performed using an automated fluorescent microscope with environmental control (CO_2_, O_2_, and humidity) (ImageXpress Micro High Content Screening System, Molecular Devices) and Image analysis software (MetaXpress Analysis software and custom modules) for the quantification of the signal intensity after staining for phospho-SMAD3 (pSMAD3). To assure that signal accurately reflects the pSMAD3 levels in nuclei of myotubes, we exploited a standard “mask-assisted” selection of nuclear signal that has been optimized by selecting specific parameters, such as signal background and size. The intensity of the signal detected in each of the 384-well plates is laser captured and quantified by MetaXpress Analysis software or Image J software alternatively.

### Gene expression analysis

Total RNA was isolated with TRIzol or RNease Micro Kit (Qiagen) and retrotranscribed using reverse transcription reagent (Applied Biosystems). qRT-PCR was performed in a Mx3000P machine (Stratagene) using SYBR Green Master Mix. Data were normalized to the expression of GAPDH gene. Primers sequences are the following (5′–3′): GAPDH (fw-AGCCGCATCTTCTTTTGCGTCG; rev-CTTCTCCATGGTGGTGAAGACG), mMyoD (fw-AATGGCTACGACACCGCCTACTA; rev-AGATGCGCTCCACTATGCTGGACA), MYOG (fw-AATGCAGCTCTCACAGCGCCTC; rev-TCAGCCGTGAGCAGATGATCC), CKM (fw-TGGAGAAGCTCTCTGTGGAAGCTC; rev-TCCGTCATGCTCTTCAGAGGGTAGTA), BAF60C2 (fw-GCGCGCAAAGCCACGAAA; rev-ATCCGGGCTCCAGACGGCATC), OCT4 (fw-GACAGGGGGAGGGGAGGAGCTAGG; rev-CTTCCCTCCAACCAGTTGCCCCAAAC), BRACHYURYT (fw-TTGATGCAAAGGAAAGAAGTGATC; rev-AGGATGAGGATTTGCAGGTG), TGFB1 (fw-GCCTGAGGCCGACTACTA; rev-CTGTGTGTACTCTGCTTGAACT), IL6 (fw-GGTACATCCTCGACGGCATCT; rev-GTGCCTCTTTGCTGCTTTCAC), CTGF (fw-TGTGCACCGCCAAAGAT; rev-GCACGTGCACTGGTACTT), and TGFB2 (fw-TGGTGAAAGCAGAGTTCAGAG; rev-CGCTGGGTTGGAGATGTTAA).

### Electrical pacing and recording

DMD and control iPSC expressing epB-BAF60C/MyoD were seeded on 48 gold electrode wells and induced to differentiate as previously described. At d10, electrical pacing was initiated by applying a pulse train amplitudes of *f* = 1 Hz, *t*_on_ = 0.5 ms (pulse length), and *V*_app_ = {2.2 V} using xCELLigence® RTCA CardioECR (ACEA eBiosciences). Undifferentiated iPSC were used as a negative control, being non-excitable cells.

### Statistics

Statistical analyses were carried out using Excel or Prism v8. Data are represented as mean ± SEM or mean ± SD as described in the figure legend. Graphs are prepared using Prism. Double tail *t* test was used for statistical analysis, *p < 0.05, **p < 0.01, and ***p < 0.001.

## Supplementary information

**Additional file 1: Figure S1**. Myogenic potential of Control and DMD iPSC expressing inducible epB vectors for BAF60C and MyoD.

**Additional file 2: Figure S2**. Validation of the exacerbated TGFβ response in DMD iPSC ex45del- derived myotubes.

**Additional file 3: Figure S3**. Optimization of parameters and antibodies used in HCS.

**Additional file 4: Figure S4**. Pharmacological inhibition of SMAD signaling.

**Additional file 5: Figure S5**. Pro-fibrotic gene activation in differentiated human myoblasts following electrical pacing.

## Abbreviations

DMD: Duchenne muscular dystrophy
HCS: High-content screening
hESC: Human embryonic stem cells
iPSC: Induced pluripotent stem cells
pSMAD: Phosphorylated SMAD

## References

[1] Sicinski P, Geng Y, Ryder-Cook AS, Barnard EA, Darlison MG, Barnard PJ. The molecular basis of muscular dystrophy in the mdx mouse: a point mutation. Science. 1989;244(4912):1578–1580. http://www.ncbi.nlm.nih.gov/pubmed/2662404. Accessed 16 Oct 2018. 10.1126/science.2662404 2662404

[2] Barberi T, Bradbury M, Dincer Z, Panagiotakos G, Socci ND, Studer L (2007). Derivation of engraftable skeletal myoblasts from human embryonic stem cells. Nat Med.

[3] Choi IY, Lim HT, Estrellas K (2016). Concordant but varied phenotypes among Duchenne muscular dystrophy patient-specific myoblasts derived using a human iPSC-based model. Cell Rep.

[4] Chal J, Oginuma M, Al Tanoury Z (2015). Differentiation of pluripotent stem cells to muscle fiber to model Duchenne muscular dystrophy. Nat Biotechnol.

[5] Shelton M, Metz J, Liu J (2014). Derivation and expansion of PAX7-positive muscle progenitors from human and mouse embryonic stem cells. Stem Cell Reports.

[6] Rao L, Qian Y, Khodabukus A, Ribar T, Bursac N (2018). Engineering human pluripotent stem cells into a functional skeletal muscle tissue. Nat Commun.

[7] Maffioletti SM, Gerli MFM, Ragazzi M (2015). Efficient derivation and inducible differentiation of expandable skeletal myogenic cells from human ES and patient-specific iPS cells. Nat Protoc.

[8] Darabi R, Arpke RW, Irion S (2012). Human ES- and iPS-derived myogenic progenitors restore DYSTROPHIN and improve contractility upon transplantation in dystrophic mice. Cell Stem Cell.

[9] Goudenege S, Lebel C, Huot NB (2012). Myoblasts derived from normal hESCs and dystrophic hiPSCs efficiently fuse with existing muscle fibers following transplantation. Mol Ther.

[10] Tanaka A, Woltjen K, Miyake K (2013). Efficient and reproducible myogenic differentiation from human iPS cells: prospects for modeling Miyoshi myopathy in vitro. PLoS One.

[11] Shoji E, Sakurai H, Nishino T (2015). Early pathogenesis of Duchenne muscular dystrophy modelled in patient-derived human induced pluripotent stem cells. Sci Rep.

[12] Davis RL, Weintraub H, Lassar AB (1987). Expression of a single transfected cDNA converts fibroblasts to myoblasts. Cell..

[13] Weintraub H, Tapscott SJ, Davis RL (1989). Activation of muscle-specific genes in pigment, nerve, fat, liver, and fibroblast cell lines by forced expression of MyoD. Proc Natl Acad Sci.

[14] Albini S, Coutinho P, Malecova B (2013). Epigenetic reprogramming of human embryonic stem cells into skeletal muscle cells and generation of contractile myospheres. Cell Rep.

[15] Forcales SV, Albini S, Giordani L (2012). Signal-dependent incorporation of MyoD-BAF60c into Brg1-based SWI/SNF chromatin-remodelling complex. EMBO J.

[16] Lenzi J, Pagani F, De Santis R (2016). Differentiation of control and ALS mutant human iPSCs into functional skeletal muscle cells, a tool for the study of neuromuscolar diseases. Stem Cell Res.

[17] Bernasconi P, Di Blasi C, Mora M, et al. Transforming growth factor-beta1 and fibrosis in congenital muscular dystrophies. Neuromuscul Disord 1999;9(1):28–33. http://www.ncbi.nlm.nih.gov/pubmed/10063832. Accessed 11 Oct 2019. 10.1016/s0960-8966(98)00093-5 10063832

[18] Hata A, Chen Y-G (2016). TGF-β signaling from receptors to Smads. Cold Spring Harb Perspect Biol.

[19] Pessina P, Kharraz Y, Jardí M (2015). Fibrogenic cell plasticity blunts tissue regeneration and aggravates muscular dystrophy. Stem Cell Reports.

[20] Dall’Agnese A, Caputo L, Nicoletti C, et al. Transcription factor-directed re-wiring of chromatin architecture for somatic cell nuclear reprogramming toward trans-differentiation. Mol Cell. 2019. 10.1016/j.molcel.2019.07.036. 10.1016/j.molcel.2019.07.036 PMC6842445 31519520

[21] Serrano AL, Mann CJ, Vidal B, Ardite E, Perdiguero E, Muñoz-Cánoves P (2011). Cellular and molecular mechanisms regulating fibrosis in skeletal muscle repair and disease. Curr Top Dev Biol.

[22] Wallace GQ, McNally EM (2009). Mechanisms of muscle degeneration, regeneration, and repair in the muscular dystrophies. Annu Rev Physiol.

[23] Rosenberg AS, Puig M, Nagaraju K (2015). Immune-mediated pathology in Duchenne muscular dystrophy. Sci Transl Med..

[24] Hicks MR, Hiserodt J, Paras K (2018). ERBB3 and NGFR mark a distinct skeletal muscle progenitor cell in human development and hPSCs. Nat Cell Biol.

[25] Muñoz-Cánoves P, Scheele C, Pedersen BK, Serrano AL (2013). Interleukin-6 myokine signaling in skeletal muscle: a double-edged sword?. FEBS J.

[26] Malecova B, Dall’Agnese A, Madaro L, et al. TBP/TFIID-dependent activation of myoD target genes in skeletal muscle cells. Elife. 2016;5(FEBRUARY2016). doi:10.7554/eLife.12534. 10.7554/eLife.12534 PMC4775216 26880551

[27] Rosa A, Papaioannou M, Krzspiak J, Brivanlou A (2014). miR-373 is regulated by TGFβ signaling and promotes mesendoderm differentiation in human embryonic stem cells. Dev Biol.

