# Supplementary figures and images for: Acute conversion of patient-derived Duchenne muscular dystrophy iPSC into myotubes reveals constitutive and inducible over-activation of TGFβ-dependent pro-fibrotic signaling
Source: Skelet Muscle. 2020 May 2;10:13. doi: 10.1186/s13395-020-00224-7 (PMC7195779; doi:10.1186/s13395-020-00224-7)

Figure S1

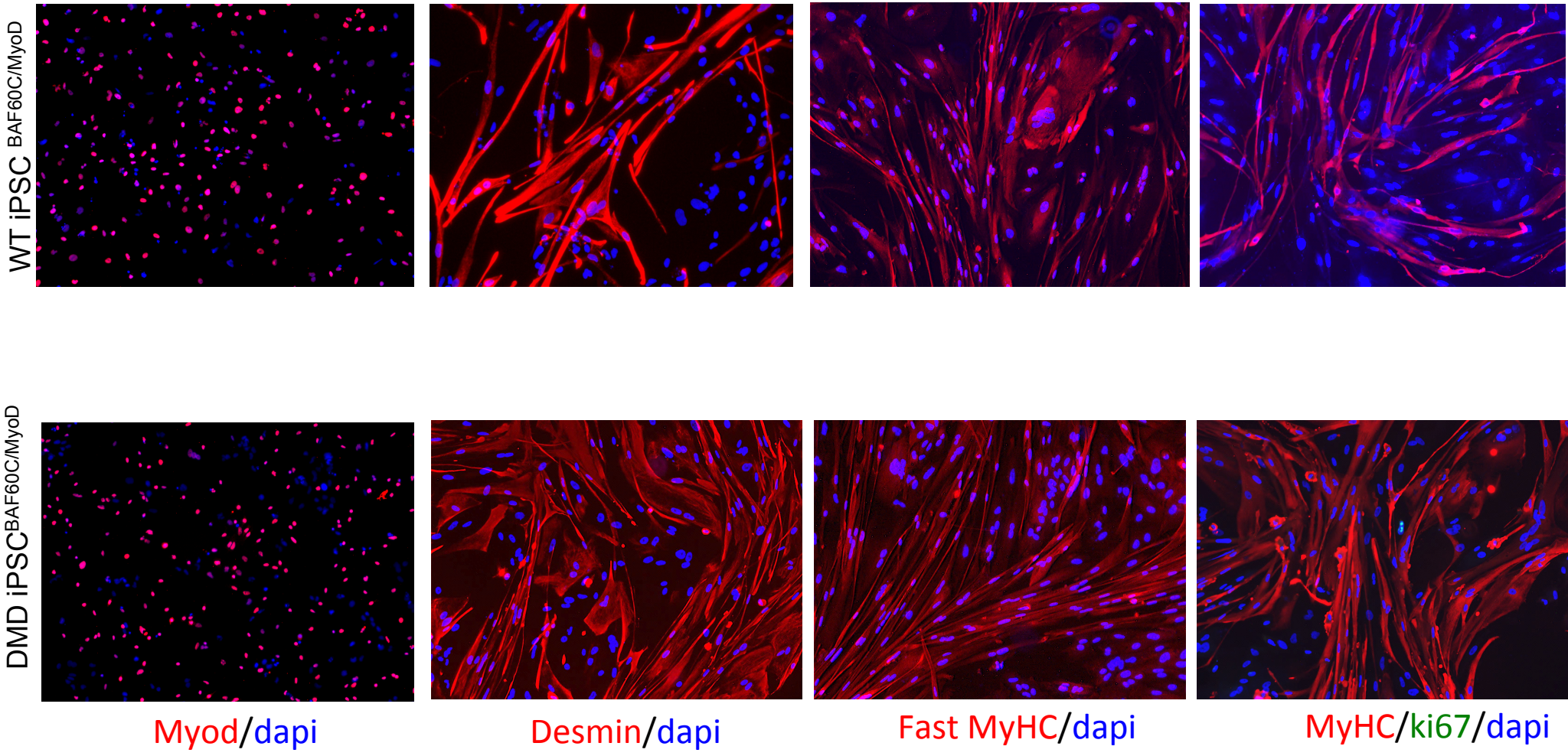

Supplement: Supplementary file 1 — Additional file 1: Figure S1. Myogenic potential of Control and DMD iPSC expressing inducible epB vectors for BAF60C and MyoD. [file 13395_2020_224_MOESM1_ESM.pdf]

Figure S2

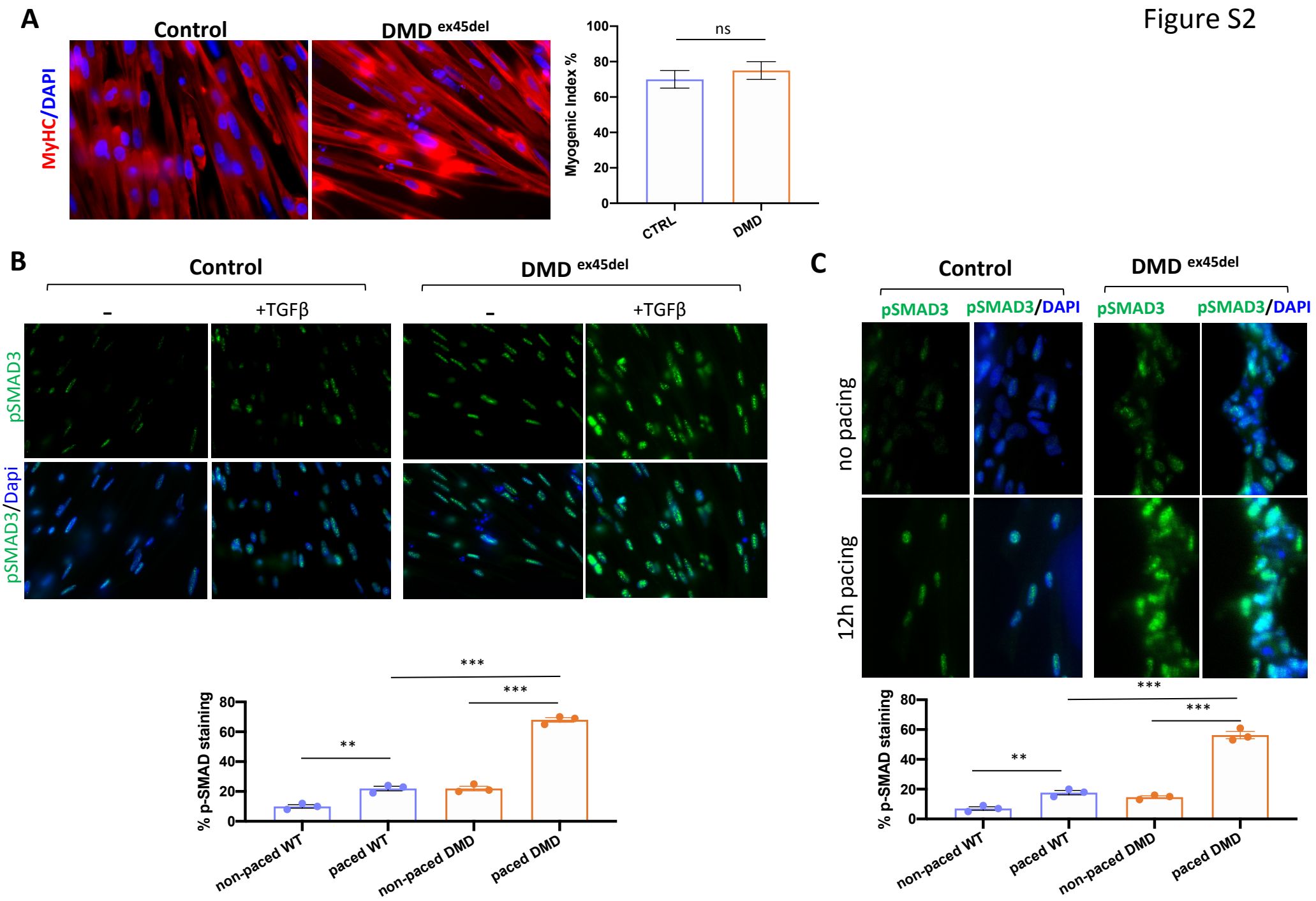

Supplement: Supplementary file 2 — Additional file 2: Figure S2. Validation of the exacerbated TGFβ response in DMD iPSC ex45del- derived myotubes. [file 13395_2020_224_MOESM2_ESM.pdf]

Figure S4

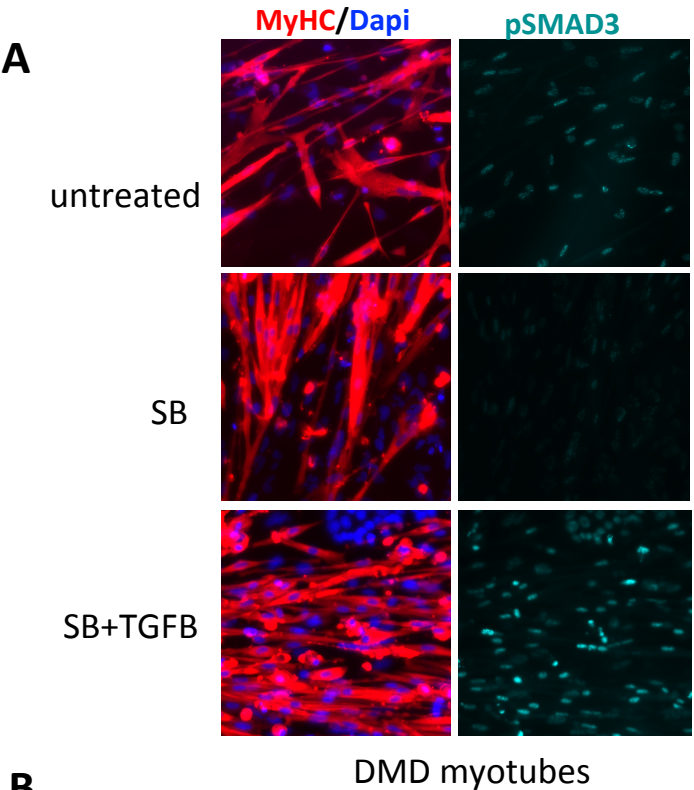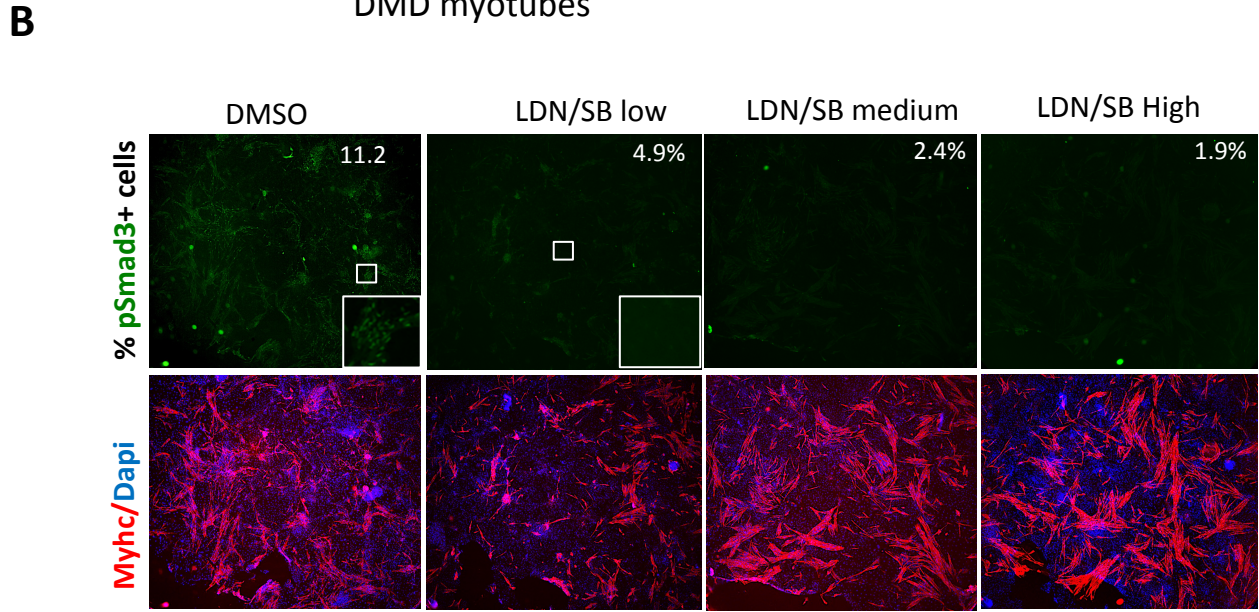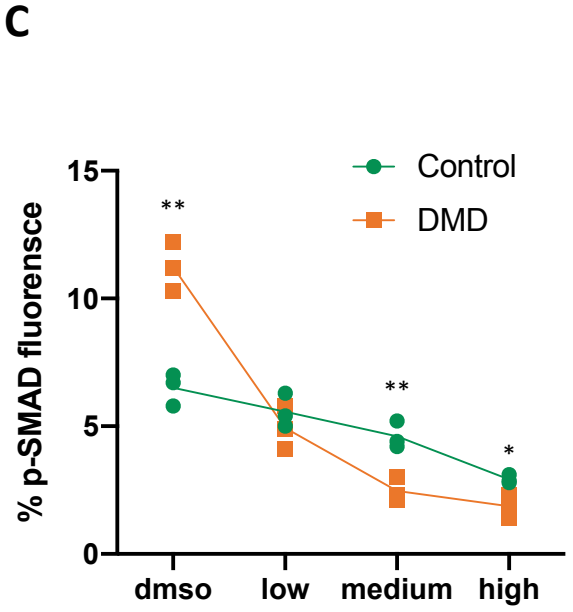

Supplement: Supplementary file 4 — Additional file 4: Figure S4. Pharmacological inhibition of SMAD signaling. [file 13395_2020_224_MOESM4_ESM.pdf]

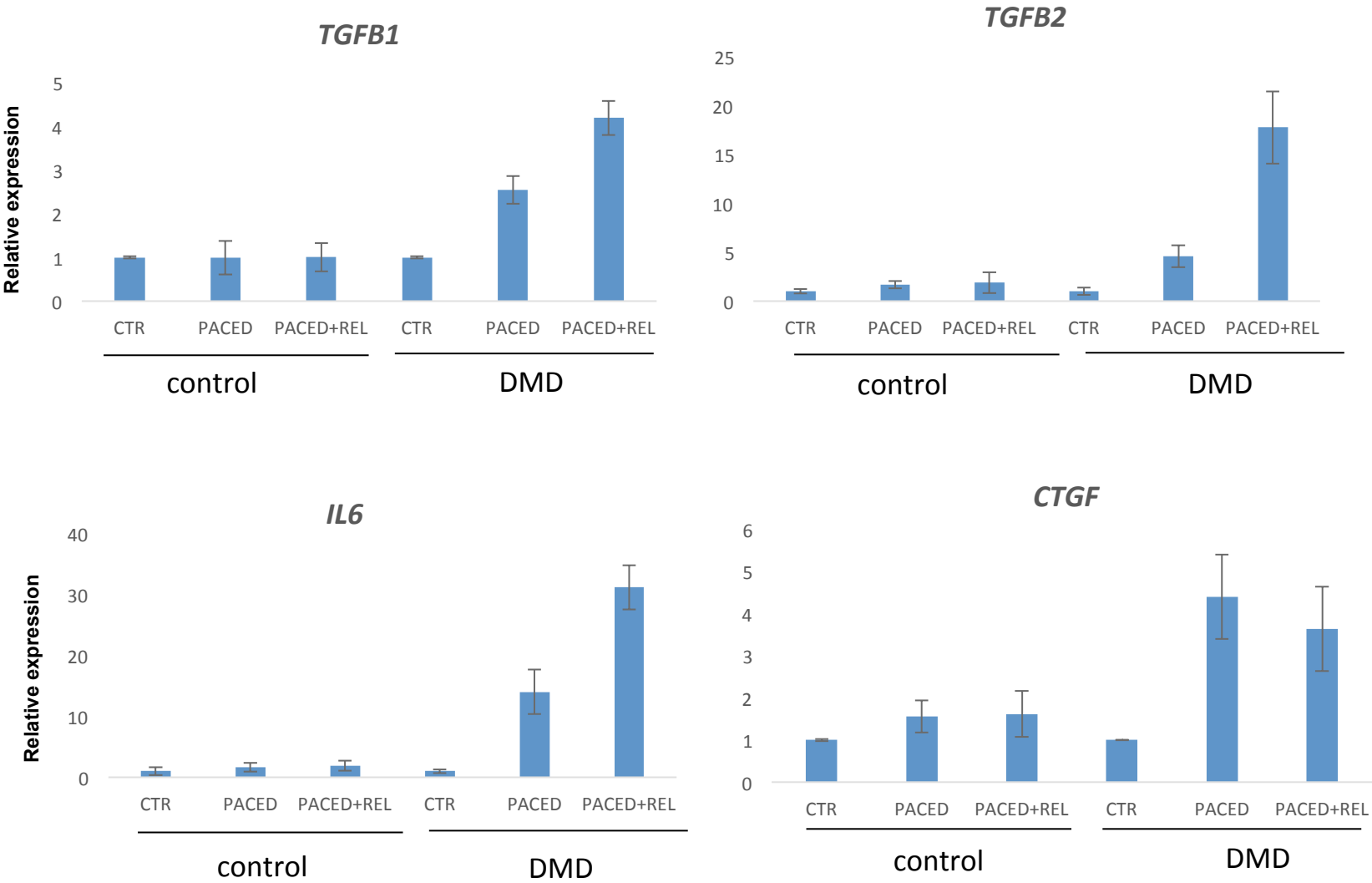

Supplement: Supplementary file 5 — Additional file 5: Figure S5. Pro-fibrotic gene activation in differentiated human myoblasts following electrical pacing. [file 13395_2020_224_MOESM5_ESM.pdf]
