# Supplementary material for: Acute conversion of patient-derived Duchenne muscular dystrophy iPSC into myotubes reveals constitutive and inducible over-activation of TGFβ-dependent pro-fibrotic signaling
Source: Skelet Muscle. 2020 May 2;10:13. doi: 10.1186/s13395-020-00224-7 (PMC7195779; doi:10.1186/s13395-020-00224-7)
Supplement: Supplementary file 3 — Additional file 3: Figure S3. Optimization of parameters and antibodies used in HCS. [file 13395_2020_224_MOESM3_ESM.pdf]

Figure S3

A

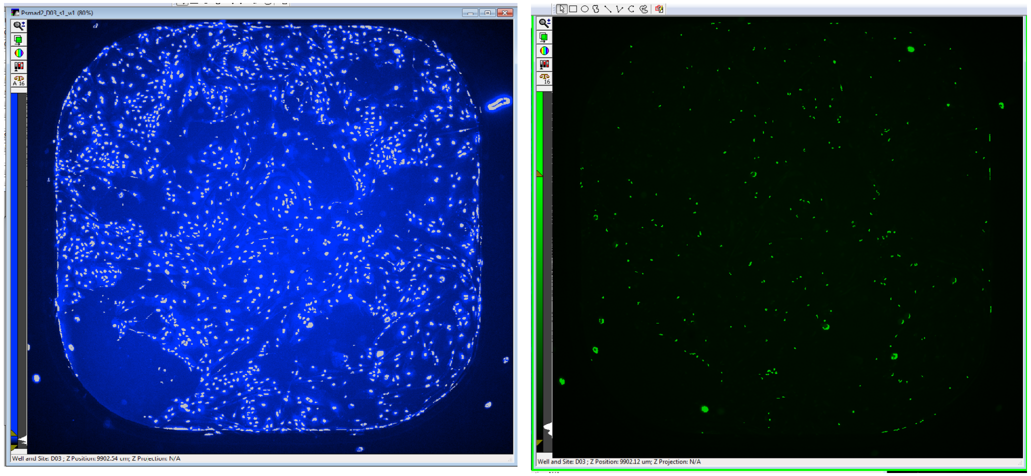

Nuclear detection

P-SMAD nuclear detection

B

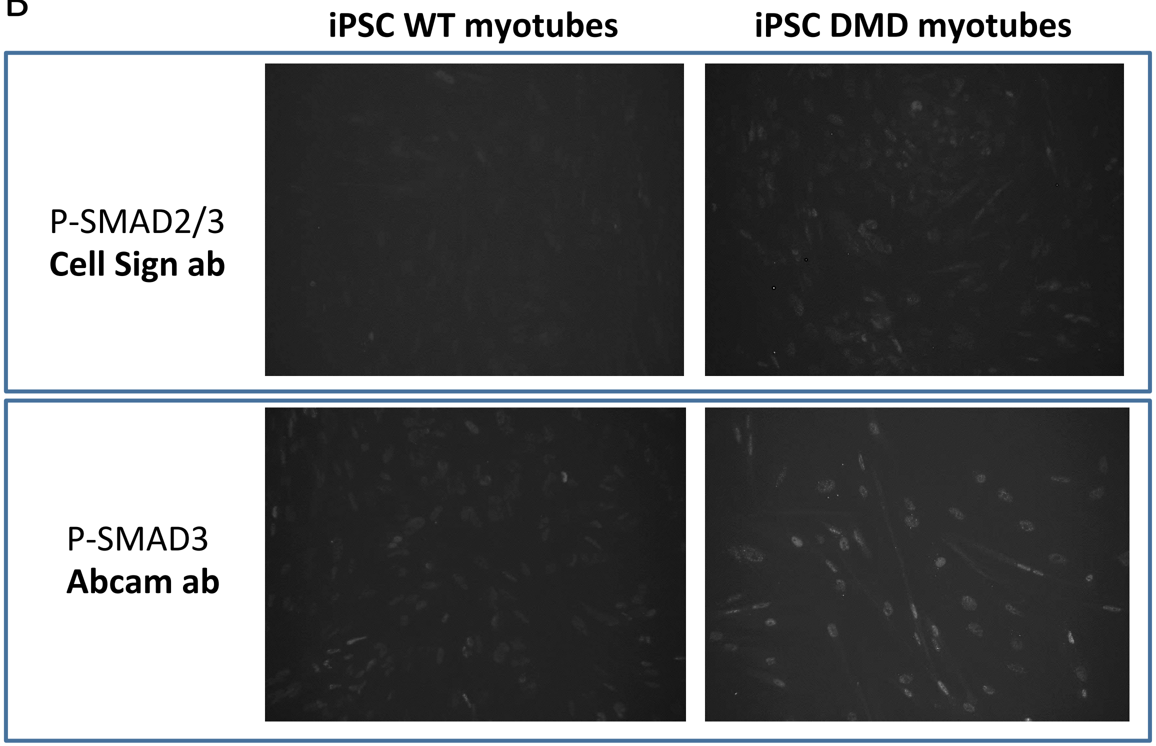

% pSMAD2/3 fluorescence

	WT	DMD
psmad CS	1,5	2,3
psmad Abcam	13,3	31
